# Endoscopic Endonasal Repair of Bilateral Choanal Atresia in a Neonate with Placement of a Steroid-Eluting Bioabsorbable Nasal Stent (PROPEL) Using a Customised Alternative Insertion Technique: A Case Report and Literature Review

**DOI:** 10.3390/jcm14238282

**Published:** 2025-11-21

**Authors:** Cosimo Galletti, Daniele Portelli, Maria Grazia Ferrisi, Fabiana Gambino, Laura Iuculano, Benedetto Sanfilippo, Gianluca Ielo, Leonard Freni, Antonino Maniaci, Francesco Ciodaro, Francesco Freni, Francesco Galletti, Bruno Galletti

**Affiliations:** 1 Department of Medicine and Surgery, University of Enna “Kore”, Piazza dell’Università, 94100 Enna, Italy; 2 Unit of Otorhinolaryngology, Department of Adult and Development Age Human Pathology “Gaetano Barresi”, University of Messina Via Consolare Valeria 1, 98125 Messina, Italy; daniele.portelli09@gmail.com (D.P.);; 3 Department of Otorhinolaryngology, University of Messina, Piazza Pugliatti 1, 98122 Messina, Italy

**Keywords:** case report, bilateral choanal atresia, nasal stent, propel, endoscopic nasal surgery

## Abstract

Introduction: Bilateral congenital choanal atresia (CCA) is a rare, life-threatening condition in neonates. This is the first reported case of PROPEL steroid-eluting stent use in a seven-day-old bilateral CCA neonate, with a customised technique developed to overcome limitations of the standard applicator. Case Presentation: A full-term male neonate presented with severe respiratory distress and was diagnosed with bilateral CCA. Endoscopic repair with mucoperiosteal flaps and drilling of the atretic plate was performed, followed by placement of a tailored PROPEL stent using a modified insertion with a Nelaton tube system. Outcomes: Recovery was uneventful. At 30 days, the stent remained well-positioned; at two, three, and six months, the neochoana was patent with no restenosis or synechiae. Conclusions: The PROPEL stent, combined with a customised insertion method, may offer a promising alternative for neonatal CCA repair. Further studies are needed to assess long-term outcomes and safety.

## 1. Introduction

Congenital choanal atresia (CCA) is a rare but critical malformation characterized by bony or membranous or both in 70% of cases, obstructing the posterior nasal airway, with an estimated incidence of approximately 1 in 5000–8000 live births, more in females. Unilateral atresia is more frequent, accounting for 60–70% of cases. In bilateral cases, neonatal obligate nasal breathing results in immediate respiratory distress, mandating prompt surgical repair [1,2,3,4,5,6,7].

Rhinofibrolaryngoscopy and CT of the skull are indispensable for a correct diagnosis and for good surgical planning [2,3,4].

A newborn affected by unilateral CCA could be asymptomatic in infancy; a bilateral type should be suspected since birth because of the signs of asphyxia and cyanosis that appear with crying [2,3]. This condition can lead to death if correct treatment and management are not undertaken.

The endoscopic transnasal choanoplasty approach has emerged as the preferred surgical method, offering direct visualization and minimal invasiveness. However, postoperative restenosis remains a frequent complication, with revision rates reaching up to 50% [6]. Traditional stenting with materials such as silicone tubes or endotracheal tubes helps preserve airway patency. Still, it is associated with complications like granulation tissue, synechiae formation, mucosal injury, and frequent need for secondary anaesthesia for stent removal [8].

Recently, bioabsorbable steroid-eluting stents have been used as an adjuvant treatment during endoscopic sinus surgery.

This particular stent is composed of bioabsorbable material (poly-D, L-lactide copolymer), which dissolves over time in the nasal mucosa. Its unique feature is the elution of corticosteroids, typically mometasone furoate, over 30 days following placement.

Another positive point is that the stent gradually dissolves over 30 days, does not require removal, eliminating the discomfort associated with the removal procedure as with traditional silicone stents. Also, the structural support provides that the healing process results are better because of the maintained patency in the nasal passage, preventing collapse of the mucoseptal flaps while tissue regeneration occurs.

The objective of this case report is to describe a novel technique for the use of the PROPEL Contour steroid-eluting bioabsorbable stent in the treatment of neonatal bilateral choanal atresia, including our innovative application system developed for the placement.

In our case, we applied the PROPEL Contour stent (Medtronic), which releases mometasone furoate, a corticosteroid with high local bioavailability that helps to minimize inflammation and the formation of granulation tissue. The stent was placed in a seven-day-old neonate following parental consent, obtained in accordance with ethical guidelines for both the application and publication of clinical information and images.

This case is reported to highlight the clinical and surgical considerations of using the PROPEL Contour stent in neonates, which may inform future management strategies for similar patients.

Our case represents the first reported instance of successful choanal atresia repair with PROPEL Contour stent placement in a seven-day-old neonate. The novelty of this report lies in the fact that in previously described cases where the PROPEL stent was used, the patients were older children. Furthermore, earlier reports did not detail the stent application system. In contrast, we developed a customised insertion technique specifically for neonates, which may serve as a guide for future applications of the PROPEL stent in this patient population.

The present case report was prepared following the CARE (CAse REport) guidelines to ensure comprehensive and transparent reporting of clinical information (see Supplementary Materials Table S1) [9].

## 2. Case Presentation

We report the case of a full-term male newborn weighing 2800 g. A timeline of the patient’s clinical course, from birth to the six-month follow-up, is provided to summarise key events and interventions. The patient had no relevant prenatal or perinatal complications, and no family history of congenital nasal malformations was reported. Immediately after birth, because of a decline in his general clinical condition and worsening dyspnoea, the patient was intubated and admitted to the neonatal intensive care unit (NICU) for respiratory distress, neonatal jaundice, and a patent foramen ovale.

An initial attempt to pass a Nelaton catheter through both nasal cavities failed, as the catheter could not be advanced. Based on the strong suspicion of bilateral choanal atresia, a flexible video rhinolaryngoscopy was performed, which revealed the presence of a membranous structure obstructing the passage into the nasopharynx. A computed tomography (CT) scan was subsequently requested to confirm the diagnosis.

**Figure 1:**
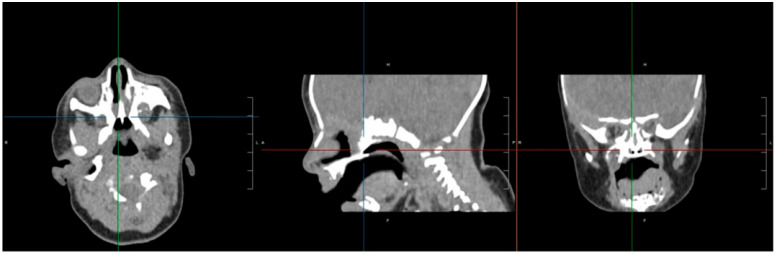
Skull CT highlights the presence of a bilateral membranous septum involving both choanae. The air column was interrupted on both sides; no bone changes were detectable, normal membranous appearance was due to incomplete physiological ossification of the planum ethmoidalis. There was no ethmoidal encephalocele. L: Left; R: right.

CT imaging confirmed bilateral choanal atresia, as demonstrated by the interruption of the air column on both sides. No bony abnormalities were detected, and the observed membranous appearance was attributed to incomplete physiological ossification of the planum ethmoidalis (Figure 1).

At seven days of life, the patient underwent transnasal endoscopic resection of the atretic plate with the formation of a neochoana, using Medtronic neuronavigation integrated with the preoperative cranial CT scan. Written informed consent for the surgery, application of the PROPEL stent, and for the publication of clinical data and images was obtained from the patient’s parents, in accordance with the ethical guidelines.

A 0°, 2.7 mm rigid endoscope was employed to perform the endoscopic surgical procedure. Tampons soaked in epinephrine diluted 1:100.000 were positioned for local vasoconstriction. The surgery was carried out in three main stages:

Creation of septal mucoperiosteal flaps: A one-sided main septal mucoperiosteal pedicled flap, known as the Hadad septal flap, was created. This latero-posteriorly based flap was fashioned using two parallel horizontal incisions starting at the anterior three-quarters of the septal mucosa, extending from its caudal end to the atretic plate. The incisions were continued posteriorly along the septum, then laterally along the atretic plate, ending at the level of the tails of the inferior and middle turbinates. This procedure was performed bilaterally.

Removal of the posterior vomer and atretic plate: The posterior third of the vomer, together with the atretic plate, was resected using a Skeeter-type drill equipped with a 2.3 mm microblade cutter. The bony resection field was deliberately made slightly larger than the overlying mucoperiosteal resection, leaving a narrow strip of mucosa attached along the border of the vomer resection to promote healing.

Drilling of the medial pterygoid plate: The medial pterygoid plate of the sphenoid bone was drilled laterally using the same Skeeter-type drill and microblade cutter to widen the posterior nasal airway.

These steps allowed for the creation of a single, wide neochoana. All raw surgical surfaces resulting from drilling or resection were covered with multiple mucosal flaps to reduce the risk of restenosis, and these flaps were secured using fibrin glue. A thin layer of fibrin glue was then applied over the entire mucosal surface of the posterior nasal cavity, the neochoana, and the rhinopharynx.

**Figure 2:**
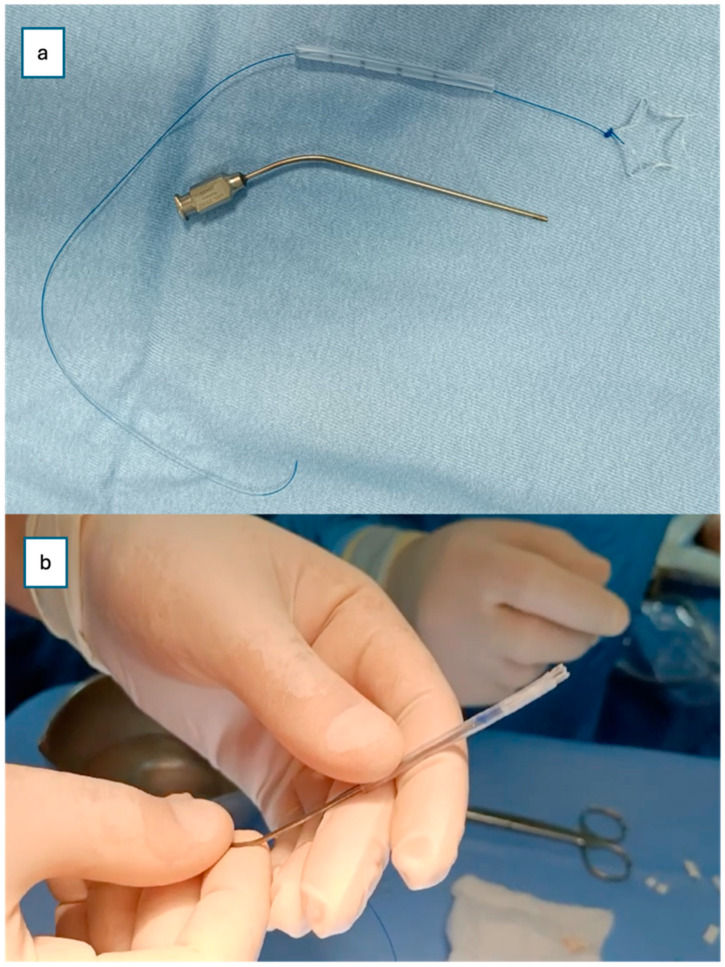
(**a**) The PROPEL Contour stent insertion system modified to allow for introduction into the nasal cavity of the newborn. (**b**) The PROPEL Contour stent folded into the Nelaton tube; an ear suction cannula was inserted to push the stent forward.

**Figure 3:**
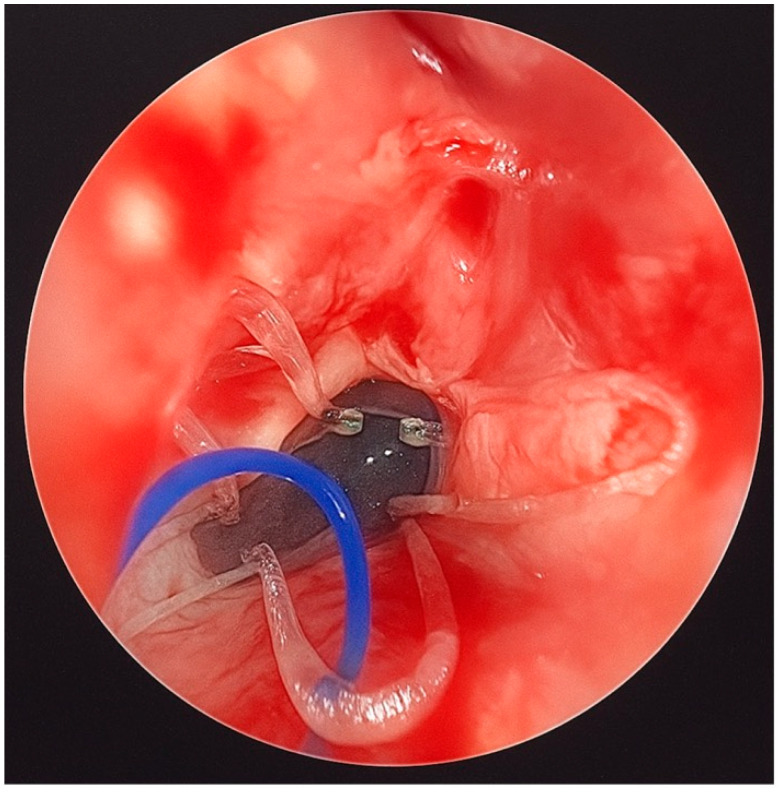
The PROPEL Contour stent was placed in the patient’s neochoana.

Finally, a steroid-eluting, bioabsorbable nasal stent (PROPEL Contour type) was tailored to fit the neochoana and placed in position (Figure 2 and Figure 3). The use of a steroid-eluting, bioabsorbable stent was chosen to maintain patency of the neochoana and reduce the risk of restenosis, as supported by previous studies in older children. As this type of stent is originally designed for placement within the frontal recess, a dedicated applicator is usually provided. However, this applicator could not be used to position the stent at the choanal level.

To overcome this limitation, an alternative placement technique was devised. A Nelaton tube, approximately 6 cm in length, was used as a delivery conduit. The stent was folded upon itself and inserted into the lumen of the Nelaton tube. It was secured with a 4-0 prolene guiding suture to prevent migration into the oropharynx and to facilitate easy removal if necessary (Figure 2).

An ear suction cannula was then inserted into the Nelaton tube to allow the surgeon to push the stent forward during deployment (Figure 2). The Nelaton tube containing the stent was advanced to the level of the choanae, while a second operator carefully pushed the suction cannula forward and simultaneously withdrew the Nelaton tube, releasing the stent precisely in position (Figure 3). The external prolene suture was secured to the skin using adhesive tape, similar to the fixation of a nasogastric tube, to prevent displacement.

The patient was returned to the NICU following surgery. Postoperatively, frequent nasal irrigation with isotonic saline solution was prescribed, along with the daily application of oily drops to maintain mucosal hydration until stent removal. A course of systemic antibiotic therapy was administered to prevent infection and support optimal healing of the surgical site. No intraoperative or postoperative complications were observed during the hospital stay or follow-up period.

**Figure 4:**
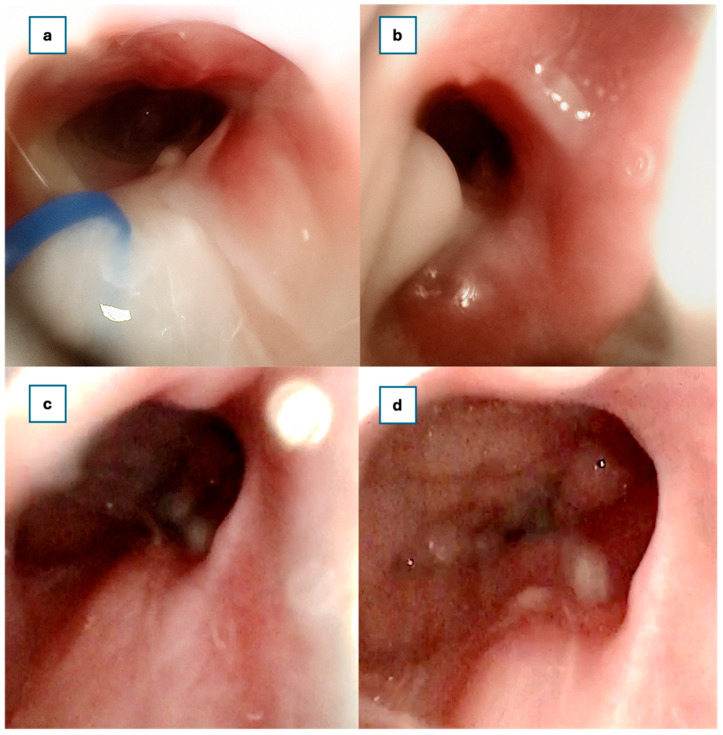
Neochoana patency. (**a**) 1st month (the PROPEL stent is still in the original position). (**b**) Neochoana patency at 2nd month. (**c**) Neochoana patency at 3rd month. (**d**) Neochoana patency at 6th month.

At 30 days postoperatively, rigid endoscopic examination confirmed the presence of a wide, patent neochoana with the steroid-eluting, bioabsorbable stent still in the correct position (Figure 4).

A two, three, and six-month follow-up examination using a rigid endoscope revealed no synechiae within the nasal cavities or nasopharynx. The patency of the neochoana was fully maintained (Figure 4).

## 3. Discussion

Restenosis remains the predominant postoperative complication after CCA repair, reported in up to 50% of cases [1,3,6]. Restenosis remains the predominant postoperative complication after CCA repair, reported in up to 50% of cases [10,11]. Traditional stents, such as silicone tubes, folded endotracheal tubes, Nelaton catheters, or Teflon splints, have been widely used to preserve airway patency. However, these materials often provoke granulation tissue, crusting, and require surgical removal or repeat anaesthesia [12,13,14,15,16].

A systematic review found that traditional endotracheal tube stent success varied widely—from 28% to 94.2%—and called for randomized controlled trials to ascertain optimal techniques, including flap use vs. stenting [8].

We report the case of a newborn with bilateral membranous choanal atresia successfully treated through endonasal endoscopic surgery followed by the placement of a steroid-eluting, bioabsorbable stent.

The use of the stent was justified by its ability to maintain postoperative neochoanal patency while providing a local, sustained release of corticosteroids to reduce mucosal inflammation and minimise the risk of restenosis, a common complication in neonatal choanal atresia repair.

In our case, we used the PROPEL Contour stent, a bioabsorbable, self-expanding implant with a PLGA backbone, tailored to fit the neochoana. This device gradually dissolves over approximately 30 to 45 days as the nasal cavity heals, maintaining patency while locally releasing 370 µg of mometasone furoate over 30 days. Detectable levels of mometasone may persist in the surrounding mucosal tissue for up to 60 days, providing sustained anti-inflammatory action and reducing the risk of restenosis without compromising mucociliary clearance [17].

Bioabsorbable steroid-eluting stents were originally developed for chronic rhinosinusitis in adults and adapted for paediatric CCA repair beginning around 2017. The stent releases corticosteroid in a controlled manner over approximately 30 days, reducing inflammation and granulation [7].

Wilcox et al. described five CCA patients (4 days to 16 years old) with steroid-eluting stents [18]; none developed restenosis over 12 months without complications [19].

Skaribas et al. (2024) reported successful management of CA restenosis using PROPEL mometasone furoate-eluting stents with no adverse events [20].

Wang et al. combined endoscopic septonasal flap creation with bioabsorbable steroid-eluting stents (Xiangtong, CFDA-approved) in 15 infants, demonstrating maintained choanal patency and flap stabilization [7].

A recent 2025 study utilized navigation-assisted endonasal U-flap techniques plus steroid-eluting stents and postoperative budesonide nebulization. Of the 26 patients, those with bioabsorbable stents achieved a 100% success rate versus 63.6% in the silicone-stented controls, with significantly fewer complications [21].

In the scientific literature, studies examining the use of the PROPEL stent in the treatment of choanal atresia are limited, but emerging evidence suggests promising outcomes.

A case series involving five paediatric patients who underwent choanal atresia surgery and were treated with PROPEL-type steroid-eluting stents showed no restenosis and excellent outcomes [18]. This suggests that steroid-eluting stents may help reduce complications seen with traditional stents, such as mucosal damage and synechiae formation [21].

Wang et al. (2021), in a retrospective study comparing bioabsorbable steroid-eluting stents and silicone stents for congenital choanal atresia repair, the bioabsorbable stents demonstrated better structural patency, faster recovery, and lower complication rates, including fewer cases of restenosis [7]. This study supports the use of steroid-eluting devices in preventing postoperative complications.

Wen et al. (2025): Another study confirmed the efficacy of steroid-eluting stents, including PROPEL, in improving long-term outcomes by reducing restenosis rates and enhancing nasal patency at 12 months of follow-up [21].

No CCA-specific complications have been reported to date with steroid-eluting stents in the literature [19]. However, broader post-market data on PROPEL (Sinus stents) highlight risks such as infection, stent migration, or oropharyngeal obstruction, though these are primarily in sinus surgery contexts [6].

An important aspect to consider, which has not been addressed in previous studies, is that the applicator provided with the device is not suitable for neonates and young children, where the nasal cavities are particularly small, making customised placement techniques essential. The standard applicator provided with the PROPEL Contour stent is specifically designed for deployment within the frontal recess, making it unsuitable for direct placement in cases of bilateral choanal atresia, where the anatomical orientation and depth differ significantly. To address this limitation, we developed a customised insertion technique. This involved loading the stent, folded on itself, into the lumen of a Nelaton tube and securing it with a prolene guiding suture to prevent migration and facilitate later removal. An ear suction device was then inserted into the Nelaton tube, allowing for controlled advancement of the stent to the choanal site. The precise positioning was achieved, ensuring stability and effectiveness without the need for the original applicator.

Despite its benefits, the PROPEL stent may not be without challenges, particularly in neonates and small infants: the device is designed for adult and paediatric sinus surgeries because the device size and fit are required to customize the stent. In our case, we tailored the stent fitting, as shown in Figure 2.

PROPEL stents are significantly more expensive than traditional silicone stents, which may limit their widespread adoption in resource-limited settings. However, in terms of biological cost for the newborn, and because of the high tax of restenosis with standard procedures, this could result in less expense than other surgical procedures with classic stents.

Although PROPEL has been well-studied in adult sinus surgery, its use in neonatal choanal atresia is relatively new, and long-term data regarding growth patterns of the nasal passages and device absorption are still needed.

In any surgical procedure, there is always the risk of improper placement, which could lead to incomplete healing or even additional complications if the stent migrates or does not maintain proper alignment.

This case highlights the feasibility and potential advantages of using a steroid-eluting, bioabsorbable stent in neonates with bilateral choanal atresia, offering a strategy to reduce restenosis and improve surgical outcomes. A major strength of this report is the detailed description of a novel stent insertion technique specifically adapted for neonates, which may guide future clinical practice. Limitations include the single-patient design and short follow-up period, which restrict the generalizability of the findings and preclude long-term assessment of nasal growth and stent absorption. Further studies involving larger cohorts and long-term follow-up are needed to confirm the safety, efficacy, and optimal application technique of PROPEL stents in neonatal choanal atresia repair.

## 4. Conclusions

This case demonstrates the feasibility and successful outcome of using a PROPEL Contour steroid-eluting bioabsorbable stent in a seven-day-old neonate with bilateral choanal atresia. The novelty of this report lies not only in the early age of the patient, but also in the development of a customised insertion technique, which may guide future applications of the stent in neonates, where standard applicators are unsuitable.

While the PROPEL stent provided effective maintenance of neochoanal patency and local corticosteroid release to reduce inflammation and prevent restenosis, several questions remain regarding its use in neonates, including long-term effects on nasal growth, optimal stent sizing, and potential complications. Larger studies, including prospective cohorts or randomized controlled trials focused on neonatal CCA, are required to validate the safety, efficacy, and best application strategies of the PROPEL stent in this population.

As clinical experience with bioabsorbable steroid-eluting stents grows, PROPEL and similar devices may become increasingly important tools in the management of paediatric airway conditions, particularly for complex congenital anomalies such as choanal atresia.

## Data Availability

The data presented in this study are available on request from the corresponding author.

## Supplementary Materials

The following supporting information can be downloaded at: https://www.mdpi.com/article/10.3390/jcm14238282/s1, Table S1: Care Checklist.
