# Supplementary material for: Endoscopic Endonasal Repair of Bilateral Choanal Atresia in a Neonate with Placement of a Steroid-Eluting Bioabsorbable Nasal Stent (PROPEL) Using a Customised Alternative Insertion Technique: A Case Report and Literature Review
Source: J Clin Med. 2025 Nov 21;14(23):8282. doi: 10.3390/jcm14238282 (PMC12693482; doi:10.3390/jcm14238282)
Supplement: Supplementary file 1 [file jcm-14-08282-s001.zip › jcm-3977662-supplementary.pdf]

CARE
case report guidelines

Table S1. CARE Checklist of information to include when writing a case report

(cc) BY-NC-ND

Topic	Item	Checklist item description	Reported on Line
**Title**	1	The diagnosis or intervention of primary focus followed by the words "case report"	1-5
**Key Words**	2	2 to 5 key words that identify diagnoses or interventions in this case report, including "case report"	23
**Abstract (no references)**	3a	Introduction: What is unique about this case and what does it add to the scientific literature?	9-11
	3b	Main symptoms and/or important clinical findings	12-13
	3c	The main diagnoses, therapeutic interventions, and outcomes	13-15
	3d	Conclusion—What is the main "take-away" lesson(s) from this case?	18-20
**Introduction**	4	One or two paragraphs summarizing why this case is unique (**may include references**)	55-57
**Patient Information**	5a	De-identified patient specific information	76
	5b	Primary concerns and symptoms of the patient	83-84
	5c	Medical, family, and psycho-social history including relevant genetic information	78-79
	5d	Relevant past interventions with outcomes	76
**Clinical Findings**	6	Describe significant physical examination (PE) and important clinical findings	83-87
**Timeline**	7	Historical and current information from this episode of care organized as a timeline	76-143
**Diagnostic Assessment**	8a	Diagnostic testing (such as PE, laboratory testing, imaging, surveys).	83-87
	8b	Diagnostic challenges (such as access to testing, financial, or cultural)	none
	8c	Diagnosis (including other diagnoses considered)	84
	8d	Prognosis (such as staging in oncology) where applicable	none
**Therapeutic Intervention**	9a	Types of therapeutic intervention (such as pharmacologic, surgical, preventive, self-care)	92-133
	9b	Administration of therapeutic intervention (such as dosage, strength, duration)	135-139
	9c	Changes in therapeutic intervention (with rationale)	none
**Follow-up and Outcomes**	10a	Clinician and patient-assessed outcomes (if available)	139-143
	10b	Important follow-up diagnostic and other test results	139-143
	10c	Intervention adherence and tolerability (How was this assessed?)	139
	10d	Adverse and unanticipated events	none
**Discussion**	11a	A scientific discussion of the strengths AND limitations associated with this case report	225-233
	11b	Discussion of the relevant medical literature **with references**	170-199
	11c	The scientific rationale for any conclusions (including assessment of possible causes)	219-221
	11d	The primary "take-away" lessons of this case report (without references) in a one paragraph conclusion	236-240
**Patient Perspective**	12	The patient should share their perspective in one to two paragraphs on the treatment(s) they received	
**Informed Consent**	13	Did the patient give informed consent? Please provide if requested	Yes X ☐ No ☐
